# Cost-utility analysis of using paliperidone palmitate in schizophrenia in China

**DOI:** 10.3389/fphar.2023.1238028

**Published:** 2023-08-03

**Authors:** Rui Luo, He Lu, Hengfen Li

**Affiliations:** ^1^ Department of Psychiatry, The First Affiliated Hospital of Zhengzhou University, Zhengzhou, Henan, China; ^2^ Department of Radiology, The People’s Hospital of Jiawang District of Xuzhou, Xuzhou, Jiangsu, China

**Keywords:** paliperidone palmitate, schizophrenia, cost-utility analysis, China, Markov

## Abstract

**Objective:** Long-acting injections (LAIs) of paliperidone palmitate have been shown to improve medication adherence and relieve psychotic symptoms. However, the specific cost-utility analysis of these LAIs in schizophrenia in China remains unclear.

**Methods:** A multi-state Markov model was constructed to simulate the economic outcomes of patients with schizophrenia in China who received paliperidone palmitate 1-month formulation (PP1M), paliperidone palmitate 3-month formulation (PP3M), and paliperidone extended-release (ER). A cost-utility analysis was conducted, mostly derived from published literature and clinical databases. All costs and utilities were discounted at a rate of 5% per annum. The primary outcome measure was the incremental cost-effectiveness ratios (ICERs). A series of sensitivity analyses were also applied.

**Results:** After 20 years, compared to ER, using PP1M resulted in an increased discounted cost from $36,252.59 to $43,207.28. This increased cost was associated with a gain in quality-adjusted life years (QALYs) from 8.60 to 9.45. As a result, the ICER for PP1M was estimated to be $8,247.46/QALY, which was lower than the willingness-to-pay (WTP) threshold of $12,756.55/QALY. When using PP3M instead of ER, the incremental cost was $768.81 and the incremental utility was 0.88 QALYs, projecting an ICER of $873.13/QALY, which was also lower than the WTP threshold of $12,756.55/QALY. The univariate sensitivity analysis showed that the costs of PP1M, PP3M, and ER had the greatest impact on ICERs. The probability sensitivity analysis (PSA) revealed that when the WTP thresholds were $12,756.55/QALY, the probability of PP1M and PP3M being cost-effective was 59.2% and 66.0%, respectively.

**Conclusion:** From the Chinese healthcare system perspective, PP3M and PP1M are both more cost-effective compared to ER, and PP3M has notable cost-utility advantages over PP1M.

## 1 Introduction

Schizophrenia is a chronic severe mental illness with high incidence, high disability rate and high recurrence rate, schizophrenia affects more than 20 million people worldwide (World Health Organization, WHO., 2022). In China, schizophrenia occurred in 0.37% of men and 0.44% of women, 0.41% of adults were disabled due to schizophrenia (Liu et al., 2014). More than 33% of patients with schizophrenia experienced more than one relapse within 1 year after discharge, which attributed to the low rate of medication adherence (Montgomery et al., 2013; Xiao et al., 2015). The potential loss of life years in Chinese patients with schizophrenia were about 18.4 years (Liu J. et al., 2022). Overall household economic burden for patients (after reimbursement) due to schizophrenia was estimated to be $2004/year, of which the costs of hospitalization accounted for 20%–99% (Xu et al., 2019; Kotzeva et al., 2023). Repeated relapses and hospitalizations placed a significant financial burden on patients and their families (Montgomery et al., 2013).

Antipsychotics are a key medication group used to relieve psychotic symptoms and improve the prognosis of patients with schizophrenia. Paliperidone extended-release (ER) is a second-generation antipsychotic (SGAS) that not only significantly reduces extrapyramidal adverse events (Davis et al., 2008; Solmi et al., 2017), but also demonstrates superior performance in reducing recurrence rates, treating negative symptoms, and improving patients’ quality of life compared to first-generation antipsychotics (FGAS) (Zhang et al., 2013; Fabrazzo et al., 2022; Schneider-Thoma et al., 2022). The emergence of long-acting injectable (LAI) paliperidone palmitate has significantly improved medication adherence among patients and reduced recurrence and rehospitalization rates without compromising clinical efficacy (Schreiner et al., 2015; Joshi et al., 2018). This advancement offers the possibility of reducing the long-term burden of patients and their families while improving the quality of life of patients (Chiou et al., 2015).

Despite the numerous advantages of LAIs, the use of paliperidone palmitate and other LAIs remains uncommon in China (Zhu et al., 2021; Sun et al., 2022; Haddad and Correll, 2023). In a large cross-sectional study of neighborhoods in 2021, less than 4% of 6,336 Chinese patients with schizophrenia agreed to use antipsychotic LAIs (Sun et al., 2022). However, in developed countries, the prescription rate for antipsychotic LAIs was as high as 18%–30% (Patel et al., 2010; Sugawara et al., 2019). This difference in usage could be attributed to the high cost of paliperidone palmitate (Yu et al., 2020). Since paliperidone has been included in the catalog of medicines covered by the national medical insurance system, with a reimbursement rate of 50%–70%, the prescription rates of paliperidone palmitate are expected to gradually increase. However, the pharmacoeconomic benefits of paliperidone palmitate remain unclear. Therefore, this study aims to evaluate the cost-utility of using paliperidone palmitate 1-month formulation (PP1M) or paliperidone palmitate 3-month formulation (PP3M) compared to ER in schizophrenia from the perspective of the Chinese healthcare system.

## 2 Materials and methods

### 2.1 Model building

**FIGURE 1:**
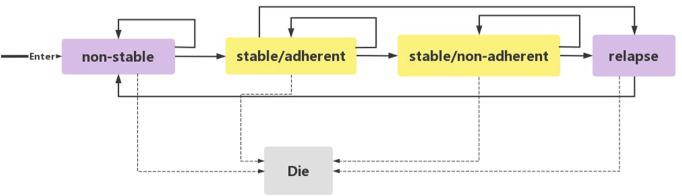
Schematic representation of the Markov model.

We used Excel 2016 to build a state-transition Markov model (Figure 1) to evaluate the cost-utility of three standard treatment options: PP1M, PP3M, and ER. This model was previously established to compare the cost-utility of lurasidone with other antipsychotics (Rajagopalan et al., 2013; Liu Y. E. et al., 2022). We set five independent health states based on the natural course of schizophrenia: 1) non-stable 2) stable/adherent 3) stable/non-adherent 4) relapse 5) death.

Acute and chronic patients were enrolled in the study. Acute patients (including patients in non-stable and relapse states) were defined as individuals diagnosed with schizophrenia according to the Diagnostic and Statistical Manual of Mental Disorders, 4th Edition (DSM-IV), with a total Positive and Negative Syndrome Scale (PANSS) score between 70 and 120 at screening and baseline, and experiencing a worsening of symptoms. Chronic patients (including patients in stable/adherent and stable/non-adherent states) were defined as individuals with a total PANSS score <70, PANSS item (P1, P2, P3, P6, P7, G8, G14) scores ≤4, and a reduction in Clinical Global Impression-Severity (CGI-S) score by ≥ 1 from open-label (OL) baseline. Patients with schizophrenia initially entered the model from the non-stable state. Following a 3-month acute phase treatment, the patients entered the stable/adherent state. Patients who did not enter this state remained in the non-stable state. Patients in the stable/adherent state entered the stable/non-adherent state if they autonomously discontinued treatment. When a patient experienced a relapsed and met clinical diagnostic criteria (DSM-IV), they entered the relapse state. From there, they reentered the model from the non-stable state. Each state in the model had the potential to transition to the death state, which was the state of absorption. Schizophrenia is a chronic disorder, and to observe the long-term prognosis and disease outcome, a 20-year simulation period was implemented in this study. Considering the onset time and the pharmacokinetics of PP1M and PP3M, a 3-month cycle was used, providing sufficient time for patients to move from one state to the next.

To reasonably simplify the model and account for the complexities of disease development and treatment processes in the real world, the study made the following assumptions: 1) All patients are in a non-stable state before entering the model. 2) The efficacy of PP1M, PP3M, and ER does not change over time. 3) The probability of each event occurring in a patient remains constant over the 20-year simulation period. 4) Patient intolerance to PP1M, PP3M, and ER was not considered. To adjust for the impact of inflation on future costs and quality-adjusted life years (QALYs), the study followed the Chinese Pharmacoeconomic Evaluation Guide 2020 (Liu et al., 2020) and used a discount rate of 5% per year. Additionally, a half-period correction method was applied to prevent the overestimation of expected survival time.

### 2.2 Simulated population

The inclusion criteria of the simulated population in this model were consistent with those of published clinical trials (Rui et al., 2014; Savitz et al., 2016): 1) adult patients aged between 18 and 70 years; 2) diagnosis of schizophrenia (according to DSM-IV); 3) baseline PANSS score between 70 and 120. The average age of the simulated population was 38 years. After a 3-week washout period, patients with schizophrenia entered the OL phase and were randomly assigned to receive either PP1M or ER. After 9 weeks of OL therapy, patients who attained a stable state were randomly assigned to receive ER, PP1M, or PP3M, and they entered a 1-year double-blind phase. According to the instructions released by Janssen, patients in the acute state could only use PP-1M for more than 4 months once they reached a stable state. Therefore, it was assumed that patients in the non-stable state could only choose between ER or PP1M.

### 2.3 Transition probability

Due to the lack of direct comparisons among PP1M, PP3M, and ER in previous studies, this study selected two randomized controlled trials with similar designs to estimate the transition probabilities of patients with schizophrenia in different states (Rui et al., 2014; Savitz et al., 2016). The all-cause discontinuation rate in the non-stable (PP1M: 28.90%; ER: 19.90%) and stable/adherent states (PP1M: 17.97%; PP3M: 16.27%; ER: 21.88%), relapse rate in stable/adherent state (PP1M:11.19%; PP3M:9.24%; ER:32.00%), and non-stable hospitalization rate (PP1M: 4.00%; PP3M: 3.00%; ER:6.00%) were obtained from related clinical trials (Rui et al., 2014; Savitz et al., 2016). The relapse rates in stable/non-adherent state for PP1M (55.11%), PP3M (52.50%), and ER (83.30%) were obtained from other published literature (Hough et al., 2010; Rui et al., 2014; Berwaerts et al., 2015). The age-dependent standard mortality rate for Chinese patients with schizophrenia was derived from the Report on China’s Cause of Death 2020 (National Center for Chronic and Noncommunicable Disease Control and Prevention, 2019) and a published study (Liu et al., 2014). To determine the transition probabilities in the model, a probability conversion formula was applied (Park et al., 2019). The formula used is as follows: r = − [ln(1—S)]/t; P = 1-e∧(-r∗T) (S represents the rate of the clinical event, t represents the duration of the clinical event, T is the cycle period, and P represents the transition probabilities). The relevant parameters of the model’s input event are shown in Table 1.

**TABLE 1:** Clinical input parameters.

Parameters	Value	Range	Distribution	References	Notes
All-cause discontinuation in non-stable state					
PP1M	0.2140	0.1926–0.2354	Beta	Savitz et al. (2016)	±10% of the mean
PP3M	0.2140	0.1926–0.2354	Beta	Savitz et al. (2016)	±10% of the mean
ER	0.2831	0.2548–0.3114	Beta	Rui et al. (2014)	±10% of the mean
All-cause discontinuation in stable/adherent state					
PP1M	0.0483	0.0435–0.0531	Beta	Savitz et al. (2016)	±10% of the mean
PP3M	0.0434	0.0391–0.0477	Beta	Savitz et al. (2016)	±10% of the mean
ER	0.1792	0.1613–0.1971	Beta	Rui et al. (2014)	±10% of the mean
Relapse in stable/adherent state					
PP1M	0.0292	0.0263–0.0321	Beta	Savitz et al. (2016)	±10% of the mean
PP3M	0.0240	0.0216–0.0264	Beta	Savitz et al. (2016)	±10% of the mean
ER	0.2655	0.2390–0.2921	Beta	Rui et al. (2014)	±10% of the mean
Relapse in stable/non-adherent state					
PP1M	0.4731	0.4258–0.5204	Beta	Hough et al. (2010)	±10% of the mean
PP3M	0.3465	0.3119–0.3812	Beta	Berwaerts et al. (2015)	±10% of the mean
ER	0.9864	0.8878–1	Beta	Rui et al. (2014)	±10% of the mean
Hospitalization in non-stable state					
PP1M	0.0102	0.0091–0.0112	Beta	Savitz et al. (2016)	±10% of the mean
PP3M	0.0076	0.0071–0.0083	Beta	Savitz et al. (2016)	±10% of the mean
ER	0.0503	0.0453–0.0554	Beta	Rui et al. (2014)	±10% of the mean
Standard mortality rate for schizophrenia					
35–39 years	0.006267			Liu et al. (2014)	Local data
40–44 years	0.004904			Liu et al. (2014)	Local data
45–49 years	0.007708			Liu et al. (2014)	Local data
50–54 years	0.007606			Liu et al. (2014)	Local data
55–59 years	0.011745			Liu et al. (2014)	Local data
60–64 years	0.035610			Liu et al. (2014)	Local data
65–78 years	0.016676			Liu et al. (2014)	Local data
Utility					
Stable state	0.23	0.21–0.25	Beta	Briggs et al. (2008)	95% CI
Non-stable state	0.15	0.14–0.17	Beta	Briggs et al. (2008)	95% CI
Cost					
PP1M	$958.87	$767.10-$1150.65	Gamma	Yaozhi.com	±20% of the mean
PP3M	$813.64	$650.91-$976.36	Gamma	Yaozhi.com	±20% of the mean
ER	$759.09	$607.27-$910.91	Gamma	Yaozhi.com	±20% of the mean
Hospitalization	$1998.90	$1599.12-$2,398.68	Gamma	Ma (2020)	±20% of the mean
Outpatient cost in stable state	$127.52	$102.02-$153.03	Gamma	Liu et al. (2022a)	±20% of the mean
Outpatient cost in non-stable state	$183.30	$164.97-$219.96	Gamma	Liu et al. (2022b)	±20% of the mean
Discounted rate	0.05	0.08		(Chinese Pharmacoeconomic Evaluation Guide 2020 (Liu et al., 2020))	Local data

### 2.4 Cost and utility

In this study, only the direct medical costs of individuals with schizophrenia were considered due to the challenges in measuring the large individual variation of indirect costs. These direct medical costs include drug, outpatient treatment, and hospitalization costs. The latest national negotiation prices in 2022, as announced by Yaozhi.com, were used to estimate the costs of the different formulations. The prices were as follows: PP1M was $2.68/mg, PP3M was $1.96/mg, and ER was $0.89/mg. The mean dose of exposure for the PP3M group was obtained from published literature as 4.61 mg per day, while for the PP1M group, it was 3.97 mg per day (Rui et al., 2014; Savitz et al., 2016). The hospitalization cost of $1,998.90 was sourced from the China Health Statistics Yearbook 2020 (Ma, X. W. 2020). The cost of outpatient examination for schizophrenia included registration fees and various examination fees obtained from published studies (Einarson et al., 2017a; Liu J. et al., 2022). In addition, previous studies have found that the cost of treating adverse reactions (ADR) accounts for less than 1% of the total cost, making a minimal contribution to the results (Einarson et al., 2017b; Arteaga et al., 2019). Therefore, the costs of ADR were not included in the model. All costs of the study were converted into US dollars using an exchange rate of US $1 = 6.72 yuan (The People’s Bank of China, 2022).

The utility values of patients with schizophrenia in different states were different. The quality of life of patients with schizophrenia in various states were converted into health utility using EQ-5D scores (Briggs et al., 2008). QALYs were used as output indicators. The Markov model’s cost and utility parameters and their distributions are shown in Table 1. The cost and utility values in the model were based on the medical consumer price index and inflated to the year 2022.

### 2.5 Outcome

The primary outcome in this study was the incremental cost-effectiveness ratio (ICER). The secondary outcomes were total costs and QALYs. ICERs represented the magnitude of increased costs in health improvement. To confirm the cost utility of the interventions, the ICER was compared to the gross domestic product (GDP) *per capita* recommended by the World Health Organization (WHO). According to the recommendations of the relevant guidelines: If ICER <3×GDP, the increased cost per QALY is considered to be acceptable and cost-effective; When ICER <1× GDP, the increased cost per QALY is completely worthwhile and very cost-effective (Liu et al., 2020). According to the National Bureau of Statistics, China’s *per capita* GDP in 2022 was $12,756.55(National Bureau of statistics of the people’s Republic of China, 2022). Therefore, the willingness-to-pay (WTP) threshold in this study was $12,756.55/QALY (National Bureau of Statistics of the People’s Republic of China, 2019).

### 2.6 Sensitivity analysis

We used univariate sensitivity analysis to assess the stability of the model when certain parameters were varied within a reasonable range and explored the factors that had the greatest impact on the ICERs in the model. The 95% confidence intervals of some parameters in the model were obtained from published literature. We assumed that the transition probability and medical cost parameters without a 95% confidence interval had a variation range of ±10% and ±20%, respectively, and the annual discount rate was within the range of 0%–8% for sensitivity analysis Chinese Pharmacoeconomic Evaluation Guide 2020. The results were presented as a tornado diagram.

To assess the uncertainty of the model, probability sensitivity analysis (PSA) was conducted using Monte Carlo simulation. The cost parameters were assumed to follow a Gamma distribution, while the utility parameters and transition probability parameters were assumed to follow a Beta distribution. The computer randomly generated the possible values according to the distribution of each parameter and calculates the ICER. This process was repeated 1,000 times. The results were presented in terms of cost-effectiveness acceptability curves (CEACs) and incremental cost-effectiveness scatter plots.

The study simulated the potential price reduction of PP1M, PP3M, and ER after the entry of generics into the market. We simulate that the costs of each drug will decrease by 11% (the current price of generic ER on the market decreased by 11% compared to the innovator drug), as well as 20%, 30%, and up to 90% in cases of centralized procurement. In addition, the actual costs of hospitalization for patients with schizophrenia at different levels of hospitals varied, so we assumed that the costs of hospitalization at ministerial, provincial, municipal, county-level, and town-level hospitals were 120%, 80%, 60%, 40%, and 20% of the mean cost, respectively. Since the median follow-up of our data source literature was 12 months, we observed the results of the time horizon for 1 year and extended the time horizon to 5, 10, 30, and 40 years to explore the impact of the length of the time horizon on the ICERs. The results were displayed in the form of a line chart.

## 3 Results

### 3.1 Cost-utility analysis

The results of the base-case analysis are shown in Table 2. After 20 years, compared to ER, the use of PP1M resulted in an increase of $6,954.69 and 0.84 QALYs, whereas the use of PP3M led to an increase of $768.81 and 0.88 QALYs. The ICERs for PP1M and PP3M were $8,247.46/QALY and $873.13/QALY, respectively, which were lower than the WTP threshold of $12,756.55/QALY, indicating that both PP1M and PP3M were more cost-effective than ER. When the model was run for 9 months, PP3M became cost-effective, and when run for 18 months, PP1M became cost-effective (Table 3). In addition, compared to PP1M, PP3M not only had lower costs but also higher utility, providing absolute pharmacoeconomic advantages.

**TABLE 2:** The results from base-case analysis.

	Total cost ($)	Total life years (QALY)	Incremental cost ($)	Incremental life years (QALY)	ICER ($/QALY)
PP1M	43207.28	9.45	6,954.69	0.84	8,247.46
PP3M	37021.40	9.48	768.81	0.88	873.13
ER	36252.59	8.60			

**TABLE 3:** Cost-effective model time horizon presented as ICER.

Circle	PP1M ($/QLAY)	PP3M ($/QLAY)
1	102,294.10	73050.24
2	36,255.48	19494.58
3	20,878.57	9,901.40
4	15,327.14	6,337.52
5	13110.24	4,764.16
6	11928.70	3,867.94

### 3.2 Sensitivity analysis

**FIGURE 2:**
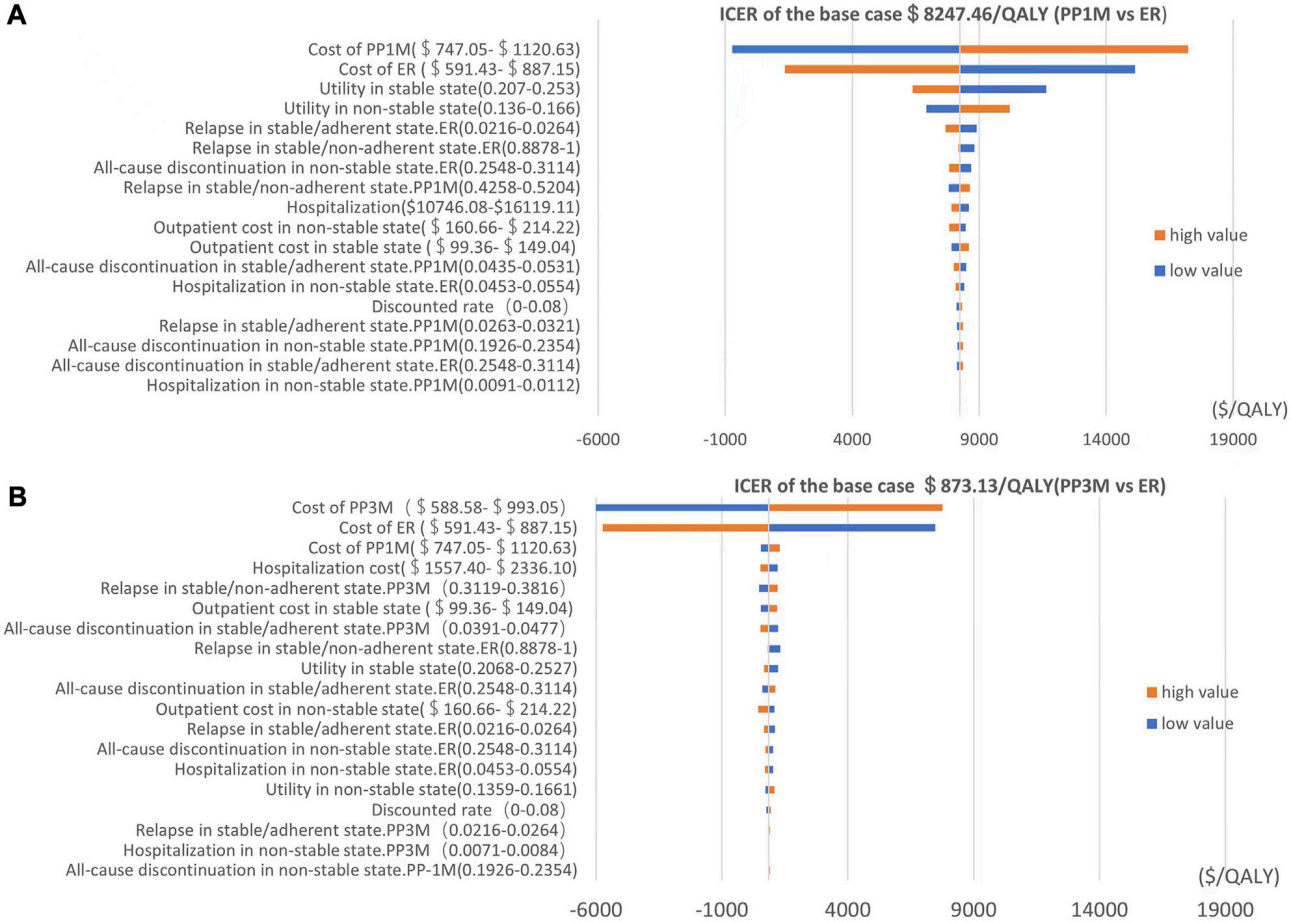
Tornado diagram showing the univariate sensitivity analysis of the Markov model simulation (red represents high values, blue represents low values). **(A)** PP1M group vs. ER group. **(B)** PP3M group vs. ER group.

We used a tornado diagram to depict the variables with the most significant impact on outcomes. Univariate sensitivity analysis showed that the costs of PP1M, PP3M, and ER had the most significant impact on the ICERs in the PP1M and PP3M groups, while other parameters had little influence on the ICERs (Figure 2).

**FIGURE 3:**
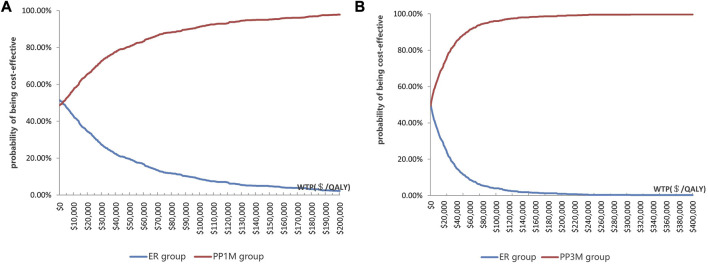
Cost-effectiveness acceptability curve showing the maximum WTP and the corresponding probability of being cost-effective. **(A)** PP1M group vs. ER group (red represents PP1M, blue represents ER). **(B)** PP3M group vs. ER group (red represents PP3M, blue represents ER).

**FIGURE 4:**
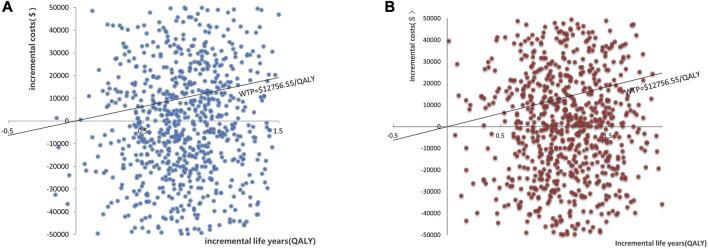
Scatter plot showing the incremental costs and incremental quality-adjusted life-year of 1,000 simulations. **(A)** PP1M group vs. ER group. **(B)** PP3M group vs. ER group.

The results of PSA are shown in Figure 3. When the WTP was $12,756.55/QALY, the probability of PP1M and PP3M being more cost-effective was 59.2% and 66.0%, respectively (Figure 3). The results of 1,000 Monte Carlo simulations are shown as a scatter plot (Figure 4). The oblique line represents the WTP threshold line, and the scatter points are mainly distributed in the first and fourth quadrants and most of them are below the WTP threshold line.

**FIGURE 5:**
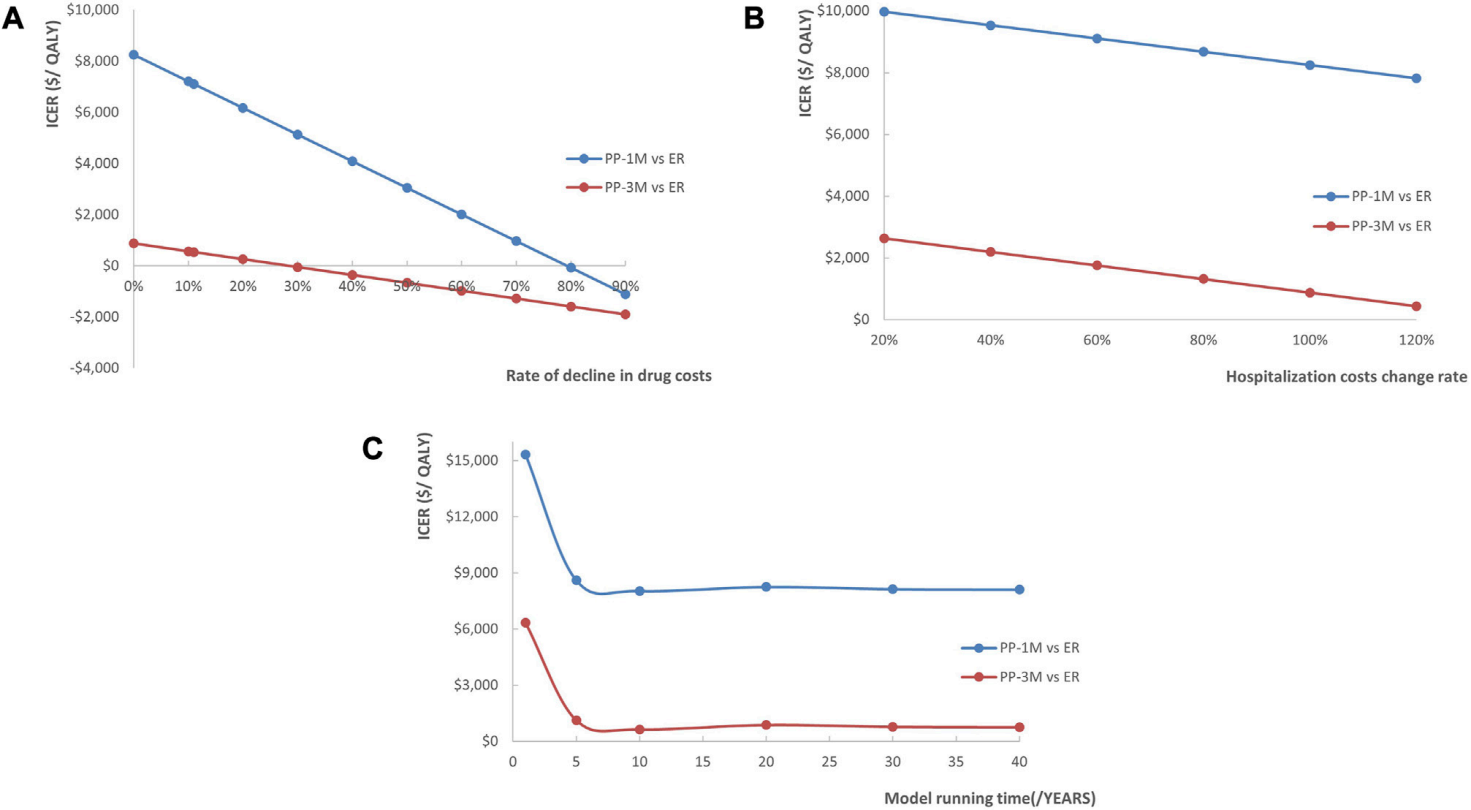
Line chart showing the changes in ICERs as **(A)** the costs of PP1M and PP3M decreased, **(B)** the costs of hospitalization increased, and **(C)** the time horizon changed (red represents PP3M vs. ER, blue represents PP1M vs. ER).

The results of the scenario analysis were presented in line graphs (Figure 5). The lower national negotiation prices of PP1M and PP3M, the higher costs of hospitalization, and the longer time horizon all contributed to more pharmacoeconomic benefits.

## 4 Discussion

This study represents the first cost-utility study of paliperidone palmitate in the treatment of schizophrenia in China. We established a Markov model to simulate the costs and outcomes associated with different treatment options for patients with schizophrenia over a 20-year study period. The study demonstrated that PP1M and PP3M were both more cost-effective than ER, with PP3M showing notable cost-utility advantages compared to PP1M. The cost-utility was consistently maintained even under rigorous sensitivity analyses. ER was more cost-effective when used for a short period (<9 months), whereas PP3M became more cost-effective after 9 months. Compared to PP1M, PP3M not only had a lower cost ($37,021.40 vs. $43207.28) but also a higher QALYs (9.48 vs. 9.45). These findings indicate that PP3M is the preferable treatment option for patients, considering its improved cost-utility profile. The results of this study hold potential implications for healthcare decision-makers in China, providing them with a solid foundation for making informed medical choices.

The univariate sensitivity analysis revealed the costs of PP1M, PP3M, and ER had the most substantial impact on the ICERs in the PP1M and PP3M groups. However, despite the impact, both PP1M and PP3M remained cost-effective, which was an expected outcome. Compared to ER, PP1M and PP3M significantly reduced the recurrence rate and improved the prognosis of patients. This suggests that considering a reasonable price range, treatments that can improve prognosis may be cost-effective. Due to drug patent protection, PP1M and PP3M were notably more expensive than ER. Considering that the price of PP1M and PP3M will decrease to a certain extent after the generic drug enters the market in the future, we conducted a scenario analysis to simulate the impact of drug centralized procurement. The analysis revealed that as the drug costs decreased, the ICERs decreased as well, and drug price reduction had a greater impact on the cost-utility of PP1M than PP3M. We also found that it was more cost-effective to use PP1M and PP3M in higher-grade hospitals. Considering that schizophrenia is a chronic disease requiring long-term medication, the length of the time horizon had a significant impact on the pharmacoeconomic benefits—the longer the time horizon, the greater the benefits. The first 5 years had the greatest impact on the ICERs. This suggests that extending the use of PP1M and PP3M would be more cost-effective, with a duration of 5 years or more being an ideal duration.

The probability of PP1M and PP3M being cost-effective was 59.2% and 66.0%, respectively. These findings are consistent with similar studies conducted in foreign countries (Einarson et al., 2017a; Einarson et al., 2017b; Arteaga et al., 2019). However, the probability that PP3M was cost-effective in similar studies conducted in France, New Zealand, and Spain was up to 99%, 77.8%, and 85.8%, respectively (Einarson et al., 2017a; Einarson et al., 2017b; Arteaga et al., 2019). These findings can be attributed to higher healthcare expenditure and higher WTP thresholds (Yu et al., 2020; Liu J. et al., 2022). Our results reflected the basic national conditions and healthcare system in China.

In our model, only the direct medical costs of patients were calculated, ignoring the indirect costs and intangible costs caused by the loss of social function of patients. However, the indirect costs of patients with schizophrenia can account for 50%–90% of the total cost, making it the main driver of the total cost (Kotzeva et al., 2023). A growing body of research has shown that long-acting antipsychotic injections can significantly improve the quality of life and prognosis of patients compared to oral medications (Gopal et al., 2020; Kim et al., 2021; Tost et al., 2023), suggesting that the actual benefits of PP1M and PP3M may be much greater than what has been observed. In addition, studies have demonstrated that PP1M and PP3M can significantly improve cognitive function (Suzuki et al., 2014; Takekita et al., 2016; Gopal et al., 2020; Tost et al., 2023), suggesting that patients with education and occupational needs may be better suited for treatment with PP1M and PP3M.

In real clinical practice, antipsychotics are commonly used in various combinations, such as combining clozapine with risperidone. The three most commonly used antipsychotics are risperidone (35.1%), olanzapine (31.3%), and clozapine (24.6%) in China (Wang et al., 2021). A pharmacoeconomic study conducted in Slovenia explored the cost-utility of risperidone, quetiapine, olanzapine, aripiprazole, and paliperidone. The study findings revealed that strategies involving aripiprazole, paliperidone, and quetiapine were dominant, while risperidone and olanzapine were the most cost-effective drugs for the treatment of acute schizophrenia (štuhec et al., 2013). Despite the lack of evidence on the pharmacoeconomic benefits of paliperidone compared to other common antipsychotics, which could impose restrictions on prescribing ER, PP1M, and PP3M in China, these medications have already been included in the reimbursement drug list (reimbursement ratio of 50%–70%). This inclusion is likely to promote the prescription of ER, PP1M, and PP3M. As a result, more cost-utility analyses of paliperidone are needed.

There were some limitations to this study. First, we selected two randomized controlled trials with similar experimental designs to obtain clinical data, which may need updating as follow-up data becomes available. Second, some parameters such as costs, transition probabilities, and utilities were derived from published literature, introducing the potential for publication bias. However, we conducted univariate sensitivity analysis and PSA to validate the stability of the model. Third, since there was no statistical difference in ADR and intolerance across different doses of paliperidone, we did not consider the cost of treatment for paliperidone intolerance and ADR. Fourth, we did not consider other real-world factors like drug combinations or potential decreases in drug therapy sensitivity following multiple relapses.

## 5 Conclusion

In summary, the use of PP1M and PP3M was cost-effective in the Chinese healthcare system. PP3M showed absolute cost-utility advantages over PP1M. Our findings will provide insights for healthcare decision-makers. However, these data do not represent the clinical situation incompletely, so more real-world studies on the cost-utility analysis of PP1M and PP3M in the Chinese population are needed in the future.

## Data Availability

The original contributions presented in the study are included in the article/Supplementary Material, further inquiries can be directed to the corresponding author.
